# Trajectories of physical, mental health and health satisfaction before and after arthritis diagnosis: a UK population-based study

**DOI:** 10.1186/s12891-025-08444-y

**Published:** 2025-03-01

**Authors:** Amy E. Taylor, Claryn S. J. Kung, Feifei Bu, Daisy Fancourt, Andrew Steptoe

**Affiliations:** Department of Behavioural Science and Health, Institute of Epidemiology & Health Care, University College London, London, UK

**Keywords:** Arthritis, Mental health, Physical functioning, Health satisfaction

## Abstract

**Background:**

Few longitudinal studies have explored changes in physical and mental health in individuals prior to and after arthritis diagnosis. This is important for understanding timing of diagnosis in relation to symptoms and their broader health impacts.

**Methods:**

Adults (≥ 16 years) reporting new diagnoses of arthritis between 2010 and 2023 in the UK Household Longitudinal Study (UKHLS) were included in the study (*N* = 5,258), along with a 1:1 matched sample of arthritis-free individuals. Trajectories of physical health (assessed using the SF-12 physical component summary), mental health (General Health Questionnaire (GHQ-12)) and satisfaction with health were constructed from 8 years prior to 8 years after diagnosis using growth curve models with linear splines. Difference in difference analysis was used to test whether changes in health measures following diagnosis were attributable to arthritis diagnosis.

**Results:**

Physical health decreased from 8 years prior to diagnosis, but began to show a steeper decline from 4 years before diagnosis. There was a small recovery in the year following diagnosis, followed by a continued decline from 4 years post diagnosis. Mental health worsened at 2 years prior to diagnosis and then remained relatively stable. Health satisfaction also decreased around 2 years prior to diagnosis, showing a gradual increase in the 3 years following diagnosis and then remaining stable. Patterns of change were similar by sex, neighbourhood deprivation and living situation. There was some evidence that changes in mental health and health satisfaction were larger and occurred earlier in individuals diagnosed at younger ages (16–49 year olds). Difference in difference models showed consistent findings, with deteriorations across all three outcomes in the arthritis group relative to their matched controls.

**Conclusions:**

Detectable changes in physical and mental health several years prior to diagnosis suggest the need to improve pathways to diagnosis. Persistence of worse mental health, particularly amongst younger people, highlights the importance of considering both physical and mental health in the years following diagnosis.

**Supplementary Information:**

The online version contains supplementary material available at 10.1186/s12891-025-08444-y.

## Introduction

Arthritis is a progressive condition characterized by joint pain, swelling, and stiffness, affecting an estimated 10 million people in the UK—roughly 15% of the population [1]. Osteoarthritis is the most common type, diagnosed in around 11% of adults in the UK [2], while rheumatoid and psoriatic arthritis are less prevalent, diagnosed in approximately 0.8% and 0.4% of adults, respectively [3, 4]. Although arthritis typically manifests in middle age, it can also begin in early adulthood or even childhood [5]. Individuals with arthritis experience difficulties with physical functioning, lower quality of life, high levels of anxiety and depression, and have been shown to be at greater risk of mortality [6–8]. Arthritis also has important economic consequences due to individuals’ reduced ability to work and the increased use of healthcare services [6, 9].

Given that the main goal of treatment for arthritis is to improve physical function and health-related quality of life [10], it is important to assess the impact of diagnosis on these aspects of wellbeing. Yet research on the wellbeing of people with arthritis has not advanced as swiftly as studies on its pathogenesis. Numerous studies have evaluated changes in physical and mental health in individuals already diagnosed with arthritis, demonstrating declining physical function (including muscle strength, mobility and self-reported disability) in the years following initial diagnosis [11–16]. One study, which recruited participants with recent-onset rheumatoid arthritis and followed them up three times over the following 12 years, allowed a closer understanding of experiences during as well as after diagnosis. It found that there was not initially a clear indication of changes in functional capacity, but this became strongly linked with severity of arthritis symptoms over time [15]. However, we still lack a comprehensive understanding of how physical and mental health change before, during and after diagnosis.

Understanding the timing and extent of these changes is important because there are known delays between symptom onset and diagnosis of arthritis, which can be particularly long for rarer arthritic conditions with earlier onset [17, 18]. These delays suggest that changes in physical and mental health are likely to be observed several months or years prior to diagnosis [18, 19]. Additionally, there is a paucity of published data on the determinants of physical and mental health outcomes among people with arthritis. Identifying factors that affect individuals' capacity to cope and adapt may help to guide strategies towards reducing some of the negative consequences of arthritis.

In this study, we used data from 13 waves of the UK Household Longitudinal Study (UKHLS) [20], to model changes in physical health, mental health, and health satisfaction before and after arthritis diagnoses. The aim of this work was to describe trajectories of these measures over time in individuals affected by arthritis and to explore whether changes over time differ by age at diagnosis, sex, socioeconomic conditions defined by neighbourhood deprivation, and living situation (whether living with a partner or not).

## Methods

### Study population

We used data from the first 13 waves (2009–2023) of the UK Household Longitudinal Study (UKHLS) [20]. UKHLS is a household panel survey of approximately 40,000 households, consisting of a nationally representative general population sample, oversampling of ethnic immigrant and ethnic minority populations and a follow up of individuals who had taken part in the British Household Panel Survey. Households are visited each year at approximately 12 month intervals and data collected via face-to-face interviewers by trained interviewers or through self-completed online questionnaires. This study used data from the adult survey completed by respondents aged 16 or older.

### Arthritis

In every wave of data collection, respondents who had not previously been interviewed were asked “Has a doctor or other health professional ever told you that you have any of these conditions?” and presented with a list of up to 20 conditions (depending on the specific wave). Arthritis was listed as a single condition in waves 1–9. From wave 10 (2018–2020) onwards, respondents were asked about rheumatoid arthritis and osteoarthritis as separate conditions. Respondents who had previously been interviewed were asked “Since [date of last interview], has a doctor or other health professional newly diagnosed you as having any of the following conditions?”, and were similarly presented with the list of conditions. Our arthritis group consisted of individuals (aged 16 or over) who reported being arthritis free when they joined the adult survey but went on to be diagnosed with arthritis at any point up until wave 13 (2021–2023). A comparison group without arthritis was sampled from the individuals (aged 16 or over) not reporting an arthritis diagnosis at any wave.

**Fig. 1:**
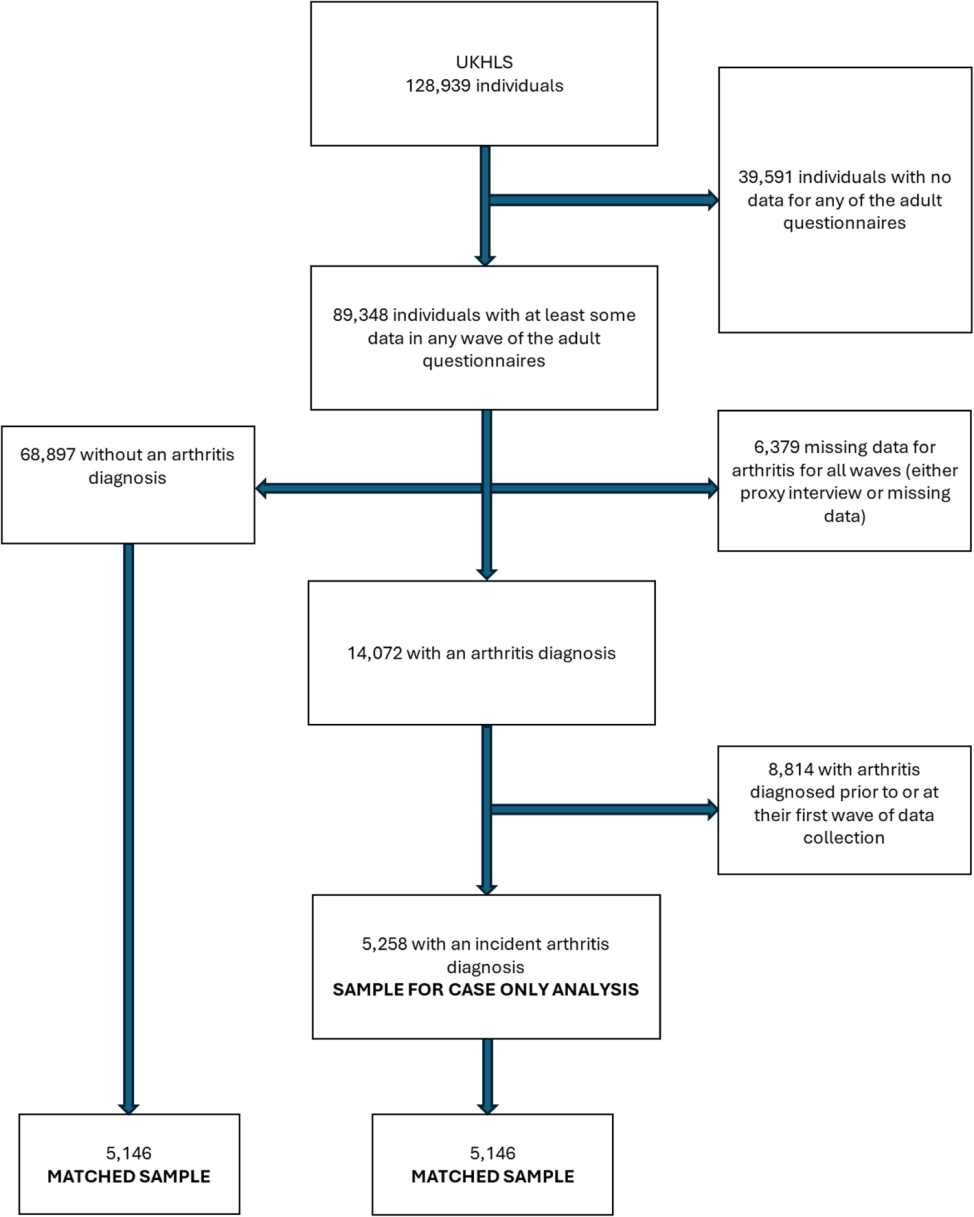
Flowchart of the study population

Of the 128,939 individuals enrolled into UKHLS, 89,348 had contributed some data to at least one wave of the adult questionnaires (16 +) (see Fig. 1). Of these, 14,072 (16%) reported a diagnosis of arthritis, but 8,814 reported their diagnosis as happening prior to or at their first wave of data collection, meaning we could not analyse experiences prior to diagnosis. This left 5,258 individuals in the analysis sample (see Fig. 1).

### Physical, mental health and health satisfaction

At each wave, the 12-Item Short Form Survey (SF-12) was administered at interview. This measures an individual’s perception of general health and the impact of health on their everyday activities, work and social activities [21]. From this survey, a physical component summary (PCS) was calculated by weighting and aggregating each item using weights derived from the US population [22]. The PCS ranges from 0 to 100 with 0 representing low functioning and 100 high functioning. The questions included in the physical component summary are shown in supplementary material.

Study participants self-completed the 12-item General Health Questionnaire (GHQ-12) at each wave [23]. The GHQ has been shown to be valid for capturing the possible presence of psychiatric disorders and compares an individual’s current mental state relative to their usual state [24]. Given the strong links between arthritis and common mental disorders [8], the GHQ-12 was selected over the mental health component of the SF-12 to measure the impact of arthritis on overall psychological burden rather than mental health related quality of life. Answers to each question were scored from 0 to 3 using the Likert scoring method, so total scores ranged from 0 to 36 with higher scores indicating greater psychological distress (see supplementary material for a list of the questions).

Participants were asked about their satisfaction with their health on a 7-point scale ranging from completely dissatisfied (lowest) to completely satisfied (highest).

### Covariates

Age (in completed years) at each wave of data collection was calculated from date of birth and date of interview. Age was categorised into the following groups: 16–49, 50–69 and 70 + . Participant sex was reported at each wave. Ethnic group was self-reported or (where self-report was unavailable) reported by a household member or derived from parental ethnicity. Highest educational qualification (Degree, A-levels, GCSEs, None) was self-reported and updated at each wave to include any new qualifications. Participants were asked about their marital status at each wave of data collection and this was recoded into a binary variable indicating whether participants lived with a partner or not: living with a partner/married or single/widowed/separated/divorced. Indices of multiple deprivation (IMD) were mapped separately for England (data from 2015), Scotland (data from 2016), Wales (data from 2014) and Northern Ireland (data from 2017) according to the Lower layer Super Output Areas that the participants lived in. IMD quintiles were combined across the countries and dichotomised into the deprived (bottom 2 quintiles) versus not deprived (top 3 quintiles) categories.

At each wave, participants were asked about whether they had ever been diagnosed with the following health conditions: asthma, congenital heart failure, coronary heart disease, angina, heart attack, stroke, emphysema, hyper or hypothyroidism, chronic bronchitis, liver conditions, cancer, diabetes, epilepsy or chronic obstructive pulmonary disease (COPD) (COPD only from wave 10 onwards). A binary indicator of any comorbidity was created indicating whether individuals had been diagnosed with any of these conditions by the time of their arthritis diagnosis.

### Statistical analyses

#### Trajectories of physical and mental health within arthritis cases

Statistical analysis was performed in Stata (Version 17). Arthritis cases were assigned a time zero at the wave when they were diagnosed. Wave of data collection was treated as the time variable, but time between waves equates to approximately 12 months for each individual. Analyses were restricted to 8 waves prior to and after time zero due to small numbers with data outside these ranges. In all models, standard errors were adjusted to account for clustering by primary sampling unit (PSU). The PSU represents small geographic areas within which households were sampled.

Trajectories of mental and physical health were assessed with growth curve models within the multilevel modelling framework [25]. We evaluated the shape of trajectories over time by fitting fractional polynomial curves up to degree two and assessing the following range of powers (−2, −1, −0.5, 0, 0.5, 1, 2, 3). This range of powers and degree of polynomial has been shown to give an adequate fit to most data [26]. Models were fitted separately for the time period before and after arthritis diagnosis. An individual level random effect was included and powers of time were allowed to vary at the individual level.

We selected the models with the highest log-likelihood and used the shape of the best fitting polynomial models (one pre-diagnosis and one post-diagnosis) to choose an approximate number of knot points for a linear spline model. We removed knots where there was no statistical evidence that the slope of adjacent splines differed from one another. We evaluated model fit by comparing observed to predicted values of the scores at each time point. Splines were entered as fixed effects but the intercept was allowed to vary at the individual level.

Models were performed unadjusted and adjusted for age at diagnosis (in categories), sex, ethnicity, education, onset wave, and having ever had a comorbidity at the time of diagnosis. To test whether the slope of the model differed by demographic characteristics, we tested interactions between model splines and age at diagnosis (16–49 years, 50–69 years and 70 + years), sex, IMD (two lowest quintiles vs highest three quintiles) and living situation (whether married/living with a partner or not).

Levels of missing data for covariates were minimal (see Table 1) so a complete case analysis was performed.

**Table 1:** Characteristics of the study population (*N* = 5,258)

		**Mean (SD)/N(%)**
Age at diagnosis	Years	58.9 (14.4)
Age category at diagnosis	16–49 50–69 70 +	1,475 (28%) 2,486 (47%) 1,297 (25%)
Sex	Male Female	1,958 (37%) 3,300 (63%)
Highest education level	Degree or above A-levels GCSEs/other None Missing	1,648 (31%) 890 (17%) 1,761 (33%) 897 (17%) 62 (1%)
Earliest recorded IMD	Not deprived (top 3 quintiles) Deprived (bottom 2 quintiles)	3,010 (57%) 2,248 (43%)
Ethnicity	White Asian African/Caribbean Other Missing	4,518 (86%) 438 (8%) 183 (3%) 117 (2%) < 5 (< 0.1%)
Marital status at diagnosis	Single Married/cohabiting Divorced/separated Widowed Missing	541 (10%) 3,470 (67%) 643 (12%) 503 (10%) 9 (0.2%)
Year of first report of arthritis	2010 2011 2012 2013 2014 2015 2016 2017 2018 2019 2020 2021 2022/3	269 (5%) 643 (12%) 517 (10%) 461 (9%) 456 (9%) 386 (7%) 385 (7%) 402 (8%) 574 (12%) 480 (10%) 265 (5%) 235 (4%) 93 (2%)
Any comorbidity ever at diagnosis^1^	Yes No	3,065 (59%) 2,101 (41%)
Cardiovascular disease ever at diagnosis^2^	Yes No	1,979 (38%) 3,187 (62%)
Diabetes ever at diagnosis	Yes No	573 (11%) 4,593 (89%)
Respiratory disease ever at diagnosis^3^	Yes No	1,011 (20%) 4,155 (80%)
Cancer ever at diagnosis	Yes No	419 (8%) 4,787 (92%)
Other chronic health condition ever at diagnosis^4^	Yes No	652 (13%) 4,554 (87%)

*IMD* Index of multiple deprivation

^1^Includes hypertension, cancer, chronic obstructive pulmonary disease, chronic bronchitis, hypothyroid, hyperthyroid, liver conditions, heart attack, stroke, diabetes, asthma, congenital heart failure, coronary heart disease, angina, emphysema, epilepsy

^2^Includes hypertension, heart attack, stroke, congenital heart failure, coronary heart disease, angina

^3^Includes chronic obstructive pulmonary disease, chronic bronchitis, emphysema, asthma

^4^Includes liver conditions, epilepsy, hypothyroid, hyperthyroid

#### Comparison of physical and mental health scores with people without arthritis

To assess if changes in mental and physical health over time were different amongst those with and without arthritis we created a matched comparison group of people without arthritis within UKHLS. Individuals with missing data on outcomes or matching variables were excluded prior to matching. We used coarsened exact matching to construct a 1:1 sample matched on year of birth (in five year intervals up until 1940, then yearly intervals until 1979, then 5 year intervals up until 2005), sex, ethnicity (white or other ethnic group), education (Degree, A-levels, GCSEs, None) and earliest IMD recorded in UKHLS (quintiles) [27]. Time zero in the comparison group was set to the same wave as the diagnosis wave for each matched arthritis case.

We computed difference in difference models which estimate within-individual changes in our outcomes of interest attributable to the diagnosis of arthritis [28]. These models calculate the difference in an outcome before and after an event (here arthritis diagnosis) within treatment and control groups and then compare the differences between groups in the two time periods. The validity of these models rests on the parallel trends assumption, where in the absence of the event, both groups would show parallel trends in the outcomes of interest. We implemented difference in difference using the xtreg command in Stata, including an interaction term between arthritis status and time period (pre/post diagnosis). We included time-varying covariates for age, year of interview, IMD, education and having ever been diagnosed with another chronic health condition. As these models assume parallel trends between groups, we set arthritis onset to be 4 years prior to diagnosis wave (based on changes observed in physical health in arthritis cases) to allow for changes in physical and mental health measures in the lead up to diagnosis.

#### Sensitivity analyses

To investigate whether results might be affected by bias due to loss to follow up, spline models were repeated restricting to individuals with data available at all 13 waves of data collection. These analyses were weighted using longitudinal weights from wave 13 provided by UKHLS to account for non- response and mortality.

## Results

The mean age at diagnosis of arthritis was 59 years (SD:14.4) and 63% of individuals with arthritis were female (Table 1). The majority of the population was of white ethnicity (86%) and most were married or living with a partner (67%). At the time of arthritis diagnosis, 59% of the study sample had been diagnosed with a comorbidity. The following number of individuals had no outcome data at any wave: 20 (0.4%) for SF-12, 38 (0.7%) for GHQ-12 and 41 (0.8%) for health satisfaction.

### Physical health

Based on the shape of the best fitting polynomials (Supplementary Figure S1), we initially used 8 knot points but reduced this to 6 (Supplementary Tables S1-S4) based on the log likelihood of the models and the lack of difference in slope between adjacent splines. Residuals from the model were normally distributed at all timepoints (see Supplementary Figure S2) and there was a good fit based on the observed and predicted values (Supplementary Table S5).

**Fig. 2:**
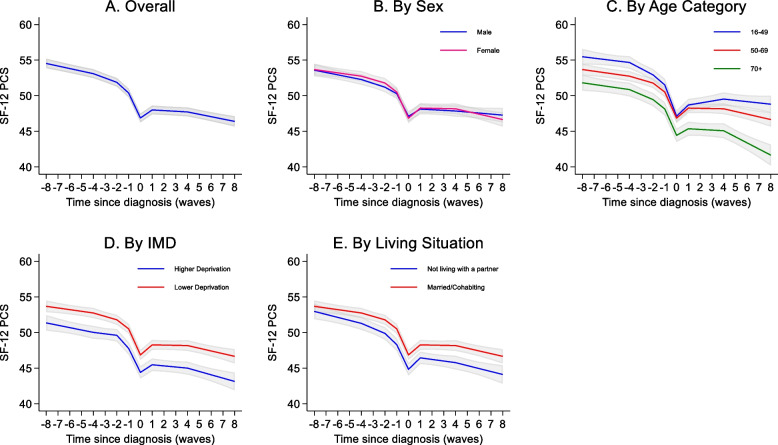
Trajectories of SF-12 physical component summary over time. For each graph, covariates apart from the one shown were set to female, 50–69 years at diagnosis, highest education level: Degree/A-level, Lower deprivation according to Indices of multiple deprivation (IMD), white ethnicity, married, no comorbidity at diagnosis. For IMD higher deprivation is the bottom 2 quintiles and lower deprivation the top 3 quintiles. On the x-axis 0 represents wave at which diagnosis was first reported, with negative numbers representing waves before and positive numbers representing waves after. Grey shading represents 95% confidence intervals

Mean physical component summary at diagnosis was 40.9 (SD: 11.9). There was a general decline in physical component summary from 8 to 4 years prior to diagnosis (−0.36 points per year, 95% CI: −0.47, −0.25), increasing to a −0.61 points per year (95% CI: −0.77, −0.45) decline between four and two years before arthritis diagnosis, a −1.49 (95% CI: −1.78, −1.20) decline between 2 and 1 years prior to diagnosis and −3.50 point decline (95% CI: −3.80, −3.21) from 1 to 0 years prior to diagnosis (Fig. 2A, Supplementary Table S4). The model indicated a small recovery in the first year after diagnosis (1.14 points, 95% CI: 0.86, 1.41) but not back up to the level observed pre-diagnosis. The general trend after this first year was a continuing decline in physical component summary over time with a similar slope four years after diagnosis to that observed several years pre diagnosis (−0.33 points per year, 95% CI: −0.45, −0.21). Unadjusted and adjusted coefficients for the splines are presented in Supplementary Tables S2 and S4.

Men and women showed similar trajectories of physical functioning over time (Fig. 2B, Supplementary Table S6). Older age was associated with lower physical functioning scores in general but the pattern of decline prior to diagnosis was similar across age groups (Fig. 2C, Supplementary Table S6). After diagnosis, there was evidence for a continued decline in physical function after the first year in the two older age groups, but not in the under 50s. Both lower deprivation and living with a partner were associated with better physical functioning but there was no strong evidence for differences in the slope of the trajectories at any time point (Fig. 2D, 2E, Supplementary Table S6).

### Mental health

Based on the shape of the best fitting polynomials (Supplementary Figure S3), we initially used 7 knot points but reduced this to 4 (Supplementary Tables S7-S10) based on the log likelihood of the models and the lack of difference in slope between adjacent splines. Model fit statistics are shown in Supplementary figure S4 and Table S11.

**Fig. 3:**
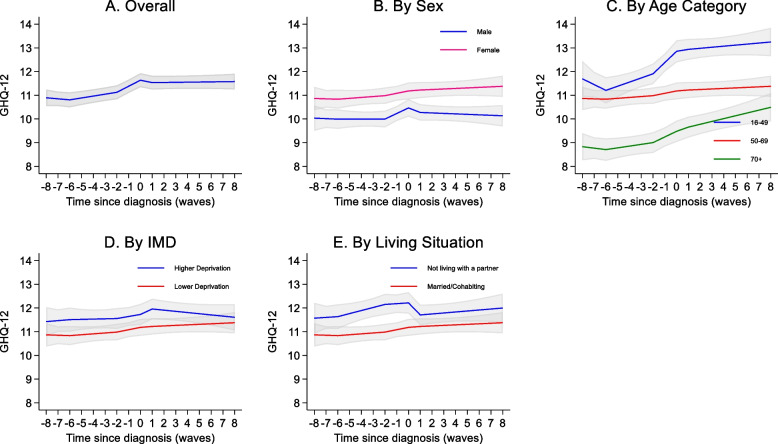
Trajectories of GHQ over time. For each graph, covariates apart from the one shown were set to female, 50–69 years at diagnosis, highest education level: Degree/A-level, Lower deprivation according to Indices of multiple deprivation (IMD), white ethnicity, married, no comorbidity at diagnosis. For IMD higher deprivation is the bottom 2 quintiles and lower deprivation the top 3 quintiles. On the x-axis 0 represents wave at which diagnosis was first reported, with negative numbers representing waves before and positive numbers representing waves after. Grey shading represents 95% confidence intervals

Mean GHQ score at diagnosis was 12.5 (SD: 6.3). The overall shape of the trajectory of GHQ over time was an initial gradual increase (indicating progressively poorer mental health) at 6 years prior to diagnosis with a steeper increase 2 years prior to diagnosis (0.25 points per year, 95% CI: 0.17, 0.34). There was no clear evidence that GHQ scores changed in the years after diagnosis, but they remained above pre-diagnosis levels (Fig. 3A, Supplementary Table S10).

On average, females had poorer GHQ scores than males (Fig. 3B, Supplementary Table S12). The slope of the increase in GHQ from 2 years prior to diagnosis and the recovery following diagnosis appeared steeper in males than in females, but there was no strong statistical evidence for interactions. Younger age groups had poorer GHQ than over 70s in general (Fig. 3C) and being between 16 and 49 at onset was associated with a bigger increase in GHQ in the 2 years prior to diagnoses than in the 50–69 age group (0.38 points per year, 95% CI: 0.13, 0.62, *p* = 0.003). There was no clear statistical evidence for differences in slope by IMD category (Fig. 3D), but there was some evidence of an interaction by living situation, with individuals living alone experiencing a decrease (improvement) in GHQ immediately following diagnosis, which was not observed among married/cohabiting individuals (−0.55 points, 95% CI:−0.90, −0.20 *p* = 0.002) (Fig. 3E).

### Health satisfaction

Based on the shape of the best fitting polynomials (Supplementary Figure S5), we initially used 8 knot points but reduced this to 4 (Supplementary Tables S13-S16). Residuals from the model were normally distributed at all timepoints (see Supplementary Figure S6) and there was a good fit based on the observed and predicted values (Supplementary Table S17).

**Fig. 4:**
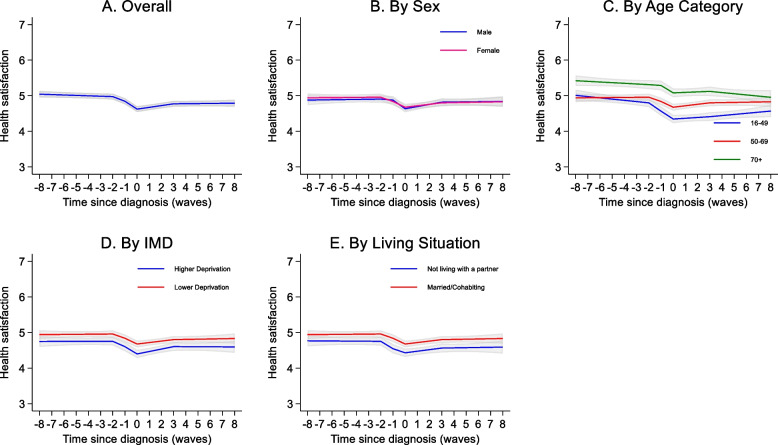
Trajectories of Health Satisfaction over time. For each graph, covariates apart from the one shown were set to female, 50–69 years at diagnosis, highest education level: Degree/A-level, Lower deprivation according to Indices of multiple deprivation (IMD), white ethnicity, married, no comorbidity at diagnosis. For IMD higher deprivation is the bottom 2 quintiles and lower deprivation the top 3 quintiles. On the x-axis 0 represents wave at which diagnosis was first reported, with negative numbers representing waves before and positive numbers representing waves after. Grey shading represents 95% confidence intervals

Mean health satisfaction score at diagnosis was 4.1 (SD: 1.7). The model indicated no change in health satisfaction over time until 2 years prior to diagnosis where there was a small decline of −0.13 (95% CI: −0.18, −0.08) points, increasing to −0.22 (95% CI: −0.27, −0.17) points from 1 year prior diagnosis. There was an initial increase in health satisfaction in the first 3 years following diagnosis (0.05 points per year, 95% CI: 0.03, 0.07) but levels remained steady after that (Fig. 4A, Supplementary Table S16).

Patterns of change in health satisfaction were similar by sex (Fig. 4B, Supplementary Table S18), IMD category (Fig. 4D) and living situation (Fig. 4E). The model indicated a steeper decline in health satisfaction at 2 years before diagnosis amongst the youngest age group compared to the oldest (−0.21 points, 95% CI:−0.36, −0.06, *p* = 0.007). The oldest age group (70 +) demonstrated a small decline in health satisfaction after 3 years which the two younger age groups did not.

### Comparison with individuals without arthritis

**Fig. 5:**
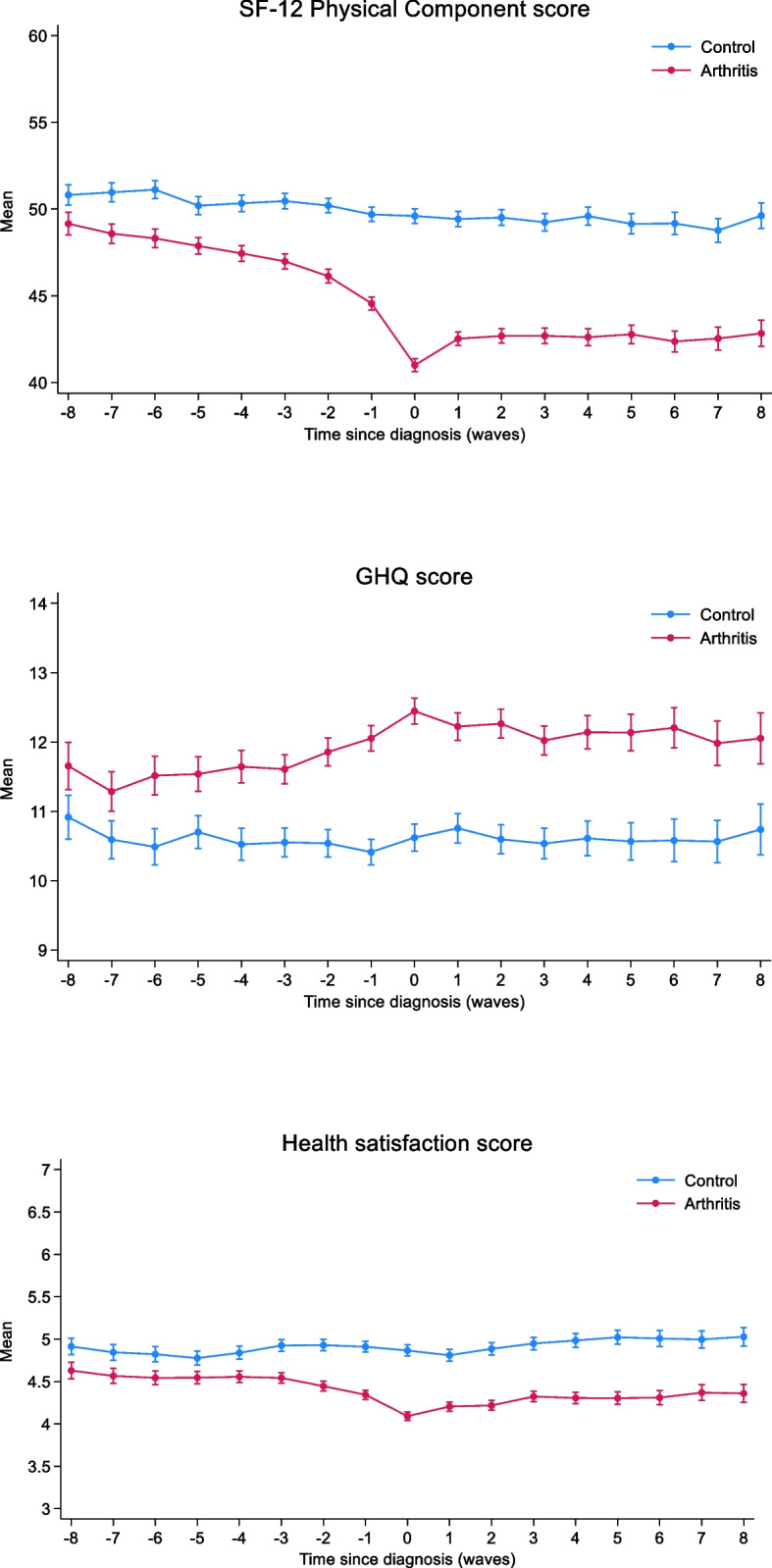
Mean SF-12 Physical component summarys, GHQ-12 Scores and Health Satisfaction in arthritis cases and matched controls. On the x-axis 0 represents wave at which diagnosis was first reported, with negative numbers representing waves before and positive numbers representing waves after. Grey shading represents 95% confidence intervals

Coarsened exact matching on year of birth, sex, ethnicity, education and IMD resulted in a sample of 5,146 individuals with arthritis and 5,146 matched controls. After matching on wave of onset, sample size for analysis was 4,789 cases and 4,789 controls. Mean values of SF-12 physical component, GHQ and health satisfaction by case control status are shown in Fig. 5. Upon visual inspection, trends appeared broadly parallel before 4 years prior to diagnosis. The unadjusted relationship between time (in waves) and each measure in controls was −0.28 points per year (95% CI: −0.32, −0.25) for SF-12 physical component summary, 0.03 points per year (95% CI: 0.01, 0.05) for GHQ-12 and −0.003 points per year (95% CI: −0.01, 0.003) for health satisfaction. There was strong evidence for differences in all three measures between cases and controls who had data at 8 years prior to diagnosis: Cases minus controls: −1.87 (95% CI: −2.68, −1.06) for SF-12, 0.67 (95% CI: 0.21, 1.12) for GHQ-12 and −0.26 (95% CI: −0.40, −0.13) for health satisfaction (all p ≤ 0.004) (See Supplementary Table S20).

Estimates from difference in difference models are shown in Table 2. There was strong evidence that individuals with arthritis showed a greater decline in physical function after 4 years pre-diagnosis of arthritis compared to the period before than controls (−2.17, 95% CI: −2.57, −1.76). They also demonstrated a greater increase in GHQ: 0.35 (95% CI: 0.12, 0.58) and a larger decline in health satisfaction: −0.17 (95% CI: −0.25, −0.10) compared to controls over the same time period.

**Table 2:** Coefficients from difference in difference models

	**N arthritis**	**N control**	**Coeff** **(Arthritis-control)**	**95% CI**	**P**
SF-12 Physical component summary	4,789	4,789	−2.17	(−2.57, −1.76)	< 0.001
GHQ Score	4,788	4,788	0.35	(0.12, 0.58)	0.002
Health satisfaction	4,789	4,789	−0.17	(−0.25, −0.10)	< 0.001

Adjusted for sex, having any comorbidity, ethnicity, IMD, year of survey, education, age. Coefficient is average difference in difference from between 8 and 4 years pre-diagnosis to between 4 years pre-diagnosis and 8 years post diagnosis

### Sensitivity analyses

The trajectories of SF-12, GHQ-12 and health satisfaction amongst individuals contributing to all waves of UKHLS (1 to 13) are shown in supplementary Figure S7 and supplementary Table S19. These showed similar patterns of change to that in the main analyses.

## Discussion

We describe patterns of change in physical and mental health prior to and following arthritis diagnosis using data from a large nationally representative sample of the adult population in the UK. Comparison with a control group without arthritis suggests that declines in physical function and mental health attributable to arthritis, are detectable several years before diagnosis and importantly, appear to persist for several years after diagnosis. Although a slight decline was detectable in the two years preceding diagnosis, health satisfaction remained relatively stable over the course of diagnosis.

We observed a decline in physical function of about 6 points (≈0.5 SD) on the SF-12 physical component summary from four years prior up until the point of diagnosis. Differences in the region of 4–5 points on the SF-12 have been shown previously between patients with and without rheumatoid arthritis or osteoarthritis and are comparable to differences in physical function observed for other chronic conditions such as angina, kidney disease and sciatica [29]. The small decline in physical function year on year from 8 to 4 years prior to diagnosis is similar in magnitude to that observed in the control group without arthritis across the time period, indicating a general age-related decline that is not specific to arthritis [30–32]. The evidence of changes as early as four years before diagnosis is observed in every demographic subgroup (age, sex, area-level deprivation and living situation) suggesting that individuals with arthritis may already be conscious of having a health problem.

It is therefore important to understand why patients are not being diagnosed sooner and whether delays are in treatment seeking or in diagnosis following contact with healthcare providers. In a sample of patients with newly presenting rheumatoid arthritis in the UK, the biggest delays were found to be from first seeing a general practitioner to being referred to a rheumatologist [18]. However, median delays were much shorter (27.2 weeks from symptom onset to rheumatology appointment) than the multi-year lag we observed in our data. This may reflect differences in delays by type of arthritis, given that osteoarthritis is much more prevalent. Indeed, in a Canadian survey of adults with chronic diseases, the average time between symptom onset and osteoarthritis diagnosis was much longer at 7.7 years [33]. It is also possible that patients experience an initial decline in physical function which they attribute to other factors or does not affect them sufficiently to warrant seeking medical advice. Given that delay in treatment following symptom onset has been shown to be associated with worse disease outcomes e.g. greater joint destruction in rheumatoid arthritis [34] and reduced opportunity for non-surgical interventions in osteoarthritis [35], this finding highlights the need for strategies to improve recognition and diagnosis of arthritis symptoms both amongst the public and in clinical practice [36]. The challenge may be in treatment seeking or health literacy—patients may not recognise symptoms or only mention some symptoms, which may prevent a doctor from making an arthritis referral.

Notably, we found evidence of a degree of recovery in physical function following diagnosis. This relief effect likely reflects an impact of treatment in halting the progression of disease and restoring some physical function or reducing pain [37, 38]. Patients have also reported that advice from healthcare professionals at diagnosis can have a positive impact on self-management of arthritis through lifestyle changes [39]. In our analyses, the decline following this immediate recovery was of similar magnitude to the downward trend observed in controls without arthritis. This is in line with data from the Health and Retirement Study, where despite a large difference in physical function between those with and without arthritis, continued age-related decline occurred at a similar rate [40]. If earlier intervention can reduce the initial decline, this may reduce the overall impact of arthritis on physical functioning.

In relation to mental health, there was evidence for worsening symptoms as assessed by the GHQ-12, from up to 6 years prior to diagnosis. However, initial declines in mental health were consistent with the small decline observed in controls over time. More marked changes in mental health occurred later than physical symptoms, from around 2 years prior to diagnosis and persisted for the 8 years after diagnosis. Average changes were relatively small (an increase of around 0.25 (≈0.04 SD) points per year just prior to diagnosis) and it is not clear the extent to which an increase of this magnitude would be clinically important. However, our finding is in line with previous research indicating a relationship between arthritis and poorer mental health. Levels of depression have been found to be twice as high amongst rheumatoid arthritis patients as in the general population and it is thought that the relationship may be bidirectional [41, 42]. Our results suggest that the impact of arthritis diagnosis on mental health may be more severe in adults diagnosed at a younger age (16–49 year olds) than in older adults. A similar pattern was seen for health satisfaction, which also declined more in the youngest age group than the older age groups just prior to diagnosis. The greater impact on younger people may reflect the perception of arthritis as a disease of older people and an inevitable part of ageing [35, 39, 43]. Furthermore, the impact on health compared to expectations of health at younger ages is likely be much more marked [44]. We are also not able to rule out the possibility of reverse causality if individuals experiencing greater changes in mental health seek medical help earlier and are therefore diagnosed at a younger age. Although health satisfaction did decline prior to diagnosis, changes were small and mean scores almost returned to pre-diagnosis levels after 3 years. It is unlikely that this is wholly due to an improvement in symptoms, given that physical functioning does not return to pre-diagnosis levels. However, diagnosis may result in better adaptation to or acceptance of health state. For example, it has been shown that perceptions patients have of their illness (lack of control, consequences of illness) may be more important than severity of disease in determining mental health and levels of pain [45].

Overall, experiences were worse for individuals living in more deprived areas, in people living alone, and (for mental health only) amongst women. This is in line with expected results. However, we did not find much evidence that any of these factors affected the trajectories, such as buffering against adverse effects. The only slight difference was in mental distress, for which people living alone had a slightly greater decrease in mental distress than people living with a partner in the years immediately following diagnosis. In previous research, spouses have been shown to play a key role in helping their partner to cope with chronic pain in arthritis, but the nature of spousal or partner support can have both positive and negative consequences on wellbeing [46]. This suggests that mental and physical health challenges of arthritis warrant attention across all demographic groups.

We observed differences in SF-12, GHQ-12 and health satisfaction between cases and controls even 8 years prior to diagnosis. There are several explanations for this. Firstly, it is possible that early symptoms of arthritis may be impacting upon health even 8 years prior to diagnosis. Secondly, these differences may reflect the impact of other health conditions; multimorbidity is common in patients with arthritis, which may be in part due to shared pathophysiological risk factors such as low-grade inflammation and metabolic dysregulation [47, 48]. Although we did adjust for presence/absence of comorbidities, we were unable to account for the severity of these conditions. Thirdly, there may be other confounding factors (e.g. sociodemographic differences) that we were unable to control for or were not adequately captured by variables in the analysis.

The key strength of this study is the use of longitudinal measures of mental and physical health captured prior to and after diagnosis of arthritis in a large representative sample. However, there are several limitations. Firstly, we were unable to distinguish between types of arthritis in the analysis. Participants of UKHLS were only asked about arthritis as a single condition up until wave 10 (2018–2020), at which point osteoarthritis and rheumatoid arthritis were reported separately (but only for new diagnoses). Therefore there were insufficient numbers to perform analyses by type of arthritis. There is evidence that there are marked differences in the course and experience of individuals with different types of arthritis [49] so these trajectories may not be generalisable to specific types of arthritis and an important next stage of this research will be to explore whether the trajectories we have identified differ in different types of arthritis. Secondly, we don’t have information on exact date of diagnosis, just the wave at which it is first reported. Therefore, it is most likely that changes in mental and physical health before arthritis diagnosis occur slightly closer to diagnosis than our model suggests and changes after, slightly further away from the exact point of diagnosis. Thirdly, there is no information on treatment following diagnosis in UKHLS so we are unable to assess if any recovery in physical or mental health is related to treatment following diagnosis. Fourthly, we had limited power to test interactions between spline models and sociodemographic factors, so we were unable to investigate interactions with more specific categories e.g. more precise age groups. Fifthly, our difference-in-differences analyses were able to account for time invariant factors (e.g. genetic predisposition to arthritis and other health conditions). However, there may have been time varying factors that we were not able to account for (e.g. access to health information and healthcare services not captured by neighbourhood deprivation levels [IMD], or other major life events), which impacts our ability to provide causal estimates of the effect of arthritis on these outcomes [28].

In conclusion, changes in physical and mental health occur several years prior to arthritis diagnosis and persist for several years following diagnosis. This highlights the opportunity for interventions aimed at cutting delays in diagnosis. Further research is needed to assess if patients can recognise early symptoms of arthritis to improve treatment seeking behaviour. Our findings of differences in mental health changes by age stresses the particular importance of support for mental health amongst younger adults diagnosed with arthritis.

## Supplementary Information

Supplementary Material 1.

## Data Availability

The data that support the findings of this study are available from Understanding Society through the UK Data Service. The data for this paper was accessed under project 237640.
