# Supplementary material for: Trajectories of physical, mental health and health satisfaction before and after arthritis diagnosis: a UK population-based study
Source: BMC Musculoskelet Disord. 2025 Mar 1;26:214. doi: 10.1186/s12891-025-08444-y (PMC11871724; doi:10.1186/s12891-025-08444-y)
Supplement: Supplementary file 1 — Supplementary Material 1. [file 12891_2025_8444_MOESM1_ESM.docx]

**Supplementary material: Trajectories of physical, mental health and health satisfaction around the time of arthritis diagnosis**

Contents

[Questions in the 12-Item Short Form Survey (SF-12) 3](#_Toc190242563)

[Questions in the General Health Questionnaire GHQ-12 5](#_Toc190242564)

[SF-12 Physical component score models 6](#_Toc190242565)

[Figure S1. Best fitting polynomial before and after diagnosis for SF-12 Physical component score 6](#_Toc190242566)

[Table S1. Unadjusted spline models with 9 splines (N=5,238) 7](#_Toc190242567)

[Table S2. Unadjusted spline models with 7 splines (N=5,238) 7](#_Toc190242568)

[Table S3. Adjusted spline models with 9 splines (N=5,177) 7](#_Toc190242569)

[Table S4. Adjusted spline models with 7 splines (N=5,177) 8](#_Toc190242570)

[Figure S2. Residuals vs fitted values and normal probability plots for SF-12 physical symptom scores at T=0, T=-8 and T=8 9](#_Toc190242571)

[Table S5. Predicted vs actual values for SF-12 physical symptom scores 10](#_Toc190242572)

[Table S6. Interactions between covariates and splines for SF-12 physical symptom scores 11](#_Toc190242573)

[GHQ-12 score models 12](#_Toc190242574)

[Table S7. Unadjusted spline models with 9 splines (N=5,220) 13](#_Toc190242575)

[Table S8. Unadjusted spline models with 5 splines (N=5,220) 13](#_Toc190242576)

[Table S9. Adjusted spline models with 8 splines (N=5,178) 13](#_Toc190242577)

[Table S10. Adjusted spline models with 5 splines (N=5,178) 14](#_Toc190242578)

[Figure S4. Residuals vs fitted values and normal probability plots for GHQ-12 scores at T=0, T=-8 and T=8 15](#_Toc190242579)

[T=0 15](#_Toc190242580)

[Table S11. Predicted vs actual values for GHQ-12 scores 16](#_Toc190242581)

[Table S12. Interactions between covariates and splines for GHQ-12 scores 17](#_Toc190242582)

[Health Satisfaction models 18](#_Toc190242583)

[Figure S5. Best fitting polynomial before and after diagnosis for Health Satisfaction 18](#_Toc190242584)

[Table S13. Unadjusted spline models with 9 splines (N=5,217) 19](#_Toc190242585)

[Table S14. Unadjusted spline models with 5 splines (N=5,239) 19](#_Toc190242586)

[Table S15. Adjusted spline models with 9 splines (N= 5,156) 19](#_Toc190242587)

[Table S16. Adjusted spline models with 5 splines (N=5,156) 20](#_Toc190242588)

[Figure S6. Residuals vs fitted values and normal probability plots for Health Satisfaction scores at T=0, T=-8 and T=8 21](#_Toc190242589)

[Table S17. Predicted vs actual values for Health Satisfaction 22](#_Toc190242590)

[Table S18. Interactions between covariates and splines for Health Satisfaction 23](#_Toc190242591)

[Figure S7. Trajectories restricted to individuals with data at all waves (1-13) of UKHLS 24](#_Toc190242592)

[Table S19. Spline models for physical component score, GHQ-12 and health satisfaction restricted to individuals with data in all waves 25](#_Toc190242593)

[Table S20. Differences in SF-12 physical component score, GHQ-12 and health satisfaction between arthritis cases and controls 8 years prior to diagnosis 26](#_Toc190242594)

# Questions in the 12-Item Short Form Survey (SF-12)

1. In general, would you say your health is:

Excellent/ Very good/Good/Fair/Poor

The following questions are about activities you might do during a typical day. Does your health now limit you in these activities? If so, how much?

2a. Moderate activities such as moving a table, pushing a vacuum cleaner, bowling, or playing golf. No, not limited at all/ Yes, limited a little/ Yes, limited a lot

2b. Climbing several flights of stairs. No, not limited at all/ Yes, limited a little/ Yes, limited a lot

3a. During the **past 4 weeks**, how much of the time have you accomplished less than you would like **as a result of your physical health**?

All of the time/Most of the time/A good bit of the time/Some of the time/A little of the time/None of the time

3b. During the **past 4 weeks**, how much of the time were you limited in the **kind** of work or other regular daily activities you do as a result of your physical health?

All of the time/Most of the time/A good bit of the time/Some of the time/A little of the time/None of the time

4a. During the **past 4 weeks**, how much of the time have you **accomplished less** than you would like **as a result of any emotional problems** (such as feeling depressed or anxious)?

All of the time/Most of the time/A good bit of the time/Some of the time/A little of the time/None of the time

During the **past 4 weeks**, how much of the time did you work or other regular daily activities **less carefully than usual as a result of any emotional problems**, such as feeling depressed or anxious?

All of the time/Most of the time/A good bit of the time/Some of the time/A little of the time/None of the time

5. During the past 4 weeks, how much did pain interfere with your normal work (including work outside the home and housework)?

Not at all/A little bit/Moderately/Quite a bit/Extremely

These questions are about how you have been feeling during the past 4 weeks. For each question, please give the one answer that comes closest to the way you have been feeling.

6a. Have you felt calm & peaceful? All of the time/Most of the time/A good bit of the time/Some of the time/A little of the time/None of the time

6b. Did you have a lot of energy? All of the time/Most of the time/A good bit of the time/Some of the time/A little of the time/None of the time

6c. Have you felt down-hearted and blue? All of the time/Most of the time/A good bit of the time/Some of the time/A little of the time/None of the time

7. During the past 4 weeks, how much of the time has your physical health or emotional problems interfered with your social activities (like visiting friends, relatives, etc.)? All of the time/Most of the time/ Some of the time/A little of the time/None of the time

# Questions in the General Health Questionnaire GHQ-12

Over the past few weeks:

1. Have you recently been able to concentrate on whatever you are doing?

2. Have you recently lost much sleep due to some worry?

3. Have you recently felt constantly under strain?

4. Have you recently felt that you could not overcome your difficulties?

5. Have you recently been feeling unhappy and depressed?

6. Have you recently been losing confidence in yourself?

7. Have you recently been thinking of yourself as a worthless person?

8. Have you recently felt that you are playing a useful role in life?

9. Have you recently felt capable of making decisions about things?

10. Have you recently been able to enjoy your normal day-to-day activities?

11. Have you recently been able to face up to your problems?

12. Have you recently been feeling reasonably happy, all things considered?

**All questions have the following options**

**0=Better than usual, 1= same as usual, 2=less than usual , 3= much less than usual**

# SF-12 Physical component score models

The best fitting polynomial prior to arthritis diagnosis was -0.5 -0.5 which uses the two terms (1/sqrt(time) and (1/sqrt(time)*ln(time) The best fitting polynomial after arthritis diagnosis was 0.5 0.5 which uses the two terms sqrt(time) and sqrt(time)*ln(time). The curves resulting from this analysis adjusted for age, sex, ethnicity, onset wave, education, ever having a comorbidity prior to diagnosis and deprivation category (IMD) are shown below.

## Figure S1. Best fitting polynomial before and after diagnosis for SF-12 Physical component score

## Table S1. Unadjusted spline models with 9 splines (N=5,238)

|  | Beta | 95% CI | |
| --- | --- | --- | --- |
| PCS 8 years pre-arthritis | 48.12 | (47.69 | 48.55) |
| **Change in PCS** |  |  |  |
| 8 - 4 years pre-arthritis | -0.35 | (-0.46 | -0.24) |
| 4 - 2 years pre-arthritis | -0.59 | (-0.75 | -0.43) |
| 2 - 1 years pre-arthritis | -1.49 | (-1.78 | -1.20) |
| 1 - 0 years pre-arthritis | -3.50 | (-3.79 | -3.20) |
| 0 - 1 years post arthritis | 1.09 | (0.80 | 1.38) |
| 1 - 2 years post arthritis | 0.08 | (-0.22 | 0.37) |
| 2 - 3 years post arthritis | -0.12 | (-0.41 | 0.17) |
| 3 - 4 years post arthritis | -0.19 | (-0.52 | 0.13) |
| 4 -8 years post arthritis | -0.29 | (-0.42 | -0.16) |

Log likelihood = -162653.2

## Table S2. Unadjusted spline models with 7 splines (N=5,238)

|  | Beta | 95% CI | |
| --- | --- | --- | --- |
| PCS 8 years pre-arthritis | 48.12 | (47.69 | 48.54) |
| **Change in PCS** |  |  |  |
| 8 - 4 years pre-arthritis | -0.35 | (-0.46 | -0.24) |
| 4 - 2 years pre-arthritis | -0.59 | (-0.75 | -0.43) |
| 2 - 1 years pre-arthritis | -1.49 | (-1.78 | -1.20) |
| 1 - 0 years pre-arthritis | -3.50 | (-3.79 | -3.20) |
| 0 - 1 years post arthritis | 1.15 | (0.88 | 1.43) |
| 1 - 4 years post arthritis | -0.08 | (-0.19 | 0.03) |
| 4 -8 post arthritis | -0.31 | (-0.44 | -0.19) |

Log likelihood = -162653.82

## Table S3. Adjusted spline models with 9 splines (N=5,177)

|  | Beta | 95% CI | |
| --- | --- | --- | --- |
| PCS 8 years pre-arthritis | 51.09 | (49.46 | 52.73) |
| **Change in PCS** |  |  |  |
| 8 - 4 years pre-arthritis | -0.36 | (-0.47 | -0.25) |
| 4 - 2 years pre-arthritis | -0.61 | (-0.77 | -0.45) |
| 2 - 1 years pre-arthritis | -1.49 | (-1.78 | -1.20) |
| 1 - 0 years pre-arthritis | -3.50 | (-3.80 | -3.21) |
| 0 - 1 years post arthritis | 1.07 | (0.78 | 1.36) |
| 1 - 2 years post arthritis | 0.05 | (-0.24 | 0.34) |
| 2 - 3 years post arthritis | -0.12 | (-0.42 | 0.17) |
| 3 - 4 years post arthritis | -0.20 | (-0.53 | 0.12) |
| 4 -8 years post arthritis | -0.31 | (-0.44 | -0.18) |

Log likelihood = -161100.62

## Table S4. Adjusted spline models with 7 splines (N=5,177)

|  | Beta | 95% CI | |
| --- | --- | --- | --- |
| PCS 8 years pre-arthritis | 51.09 | (49.46 | 52.72) |
| **Change in PCS** |  |  |  |
| 8 - 4 years pre-arthritis | -0.36 | (-0.46 | -0.25) |
| 4 - 2 years pre-arthritis | -0.61 | (-0.77 | -0.45) |
| 2 - 1 years pre-arthritis | -1.49 | (-1.78 | -1.20) |
| 1 - 0 years pre-arthritis | -3.50 | (-3.80 | -3.21) |
| 0 - 1 years post arthritis | 1.14 | (0.86 | 1.41) |
| 1 - 4 years post arthritis | -0.09 | (-0.21 | 0.02) |
| 4 -8 post arthritis | -0.33 | (-0.45 | -0.21) |

Log likelihood = -161101.18

## Figure S2. Residuals vs fitted values and normal probability plots for SF-12 physical symptom scores at T=0, T=-8 and T=8

T=0

T=-8

T=8

## Table S5. Predicted vs actual values for SF-12 physical symptom scores

|  |  | **Observed values** | | | | **Fitted values** | | | | | **Difference**  **(Fitted -observed values)** | | |
| --- | --- | --- | --- | --- | --- | --- | --- | --- | --- | --- | --- | --- | --- |
| **Time since diagnosis (waves)** | **N** | **Mean** | **SD** | **90% limits of agreement** | | **N** | **Mean** | **SD** | **90% limits of agreement** | | **N** | **Mean** | **SD** |
| -8 | 1295 | 48.8 | 10.7 | 33.0 | 59.1 | 1500 | 48.4 | 8.7 | 35.2 | 57.9 | 1288 | -0.1 | 7.0 |
| -7 | 1686 | 48.4 | 10.6 | 32.1 | 58.6 | 1895 | 48.2 | 8.7 | 34.8 | 57.7 | 1679 | 0.0 | 6.9 |
| -6 | 1965 | 48.0 | 11.1 | 31.6 | 58.8 | 2215 | 47.8 | 8.7 | 35.0 | 57.5 | 1955 | 0.0 | 6.9 |
| -5 | 2403 | 47.7 | 11.1 | 31.6 | 58.8 | 2645 | 47.5 | 8.7 | 34.8 | 57.2 | 2391 | 0.1 | 6.8 |
| -4 | 2802 | 47.3 | 11.3 | 29.4 | 58.5 | 3091 | 47.0 | 8.8 | 33.9 | 56.9 | 2791 | 0.0 | 6.7 |
| -3 | 3238 | 46.8 | 11.5 | 29.0 | 58.3 | 3578 | 46.5 | 8.9 | 33.3 | 56.5 | 3216 | -0.1 | 6.9 |
| -2 | 3772 | 46.0 | 11.6 | 28.2 | 57.8 | 4143 | 45.8 | 9.0 | 32.4 | 56.0 | 3739 | 0.1 | 6.9 |
| -1 | 4455 | 44.4 | 12.1 | 25.8 | 57.8 | 4853 | 44.2 | 9.0 | 30.9 | 54.6 | 4424 | 0.0 | 7.1 |
| 0 | 4710 | 40.9 | 11.9 | 23.5 | 55.9 | 5125 | 40.6 | 9.1 | 27.2 | 51.1 | 4659 | 0.0 | 7.2 |
| 1 | 4008 | 42.4 | 12.1 | 24.5 | 56.7 | 4329 | 42.1 | 9.0 | 28.8 | 52.4 | 3969 | 0.1 | 7.0 |
| 2 | 3609 | 42.6 | 12.0 | 24.6 | 56.6 | 3835 | 42.2 | 9.0 | 28.8 | 52.6 | 3583 | -0.1 | 6.8 |
| 3 | 3153 | 42.5 | 12.2 | 24.1 | 56.7 | 3358 | 42.2 | 9.0 | 29.0 | 52.6 | 3131 | -0.1 | 6.9 |
| 4 | 2495 | 42.5 | 12.2 | 24.3 | 56.6 | 2637 | 42.3 | 9.0 | 29.1 | 52.8 | 2478 | 0.1 | 6.8 |
| 5 | 2075 | 42.5 | 12.1 | 24.7 | 56.7 | 2183 | 42.2 | 9.0 | 28.7 | 52.6 | 2062 | -0.1 | 6.8 |
| 6 | 1660 | 42.2 | 12.1 | 23.9 | 56.2 | 1754 | 42.2 | 9.1 | 28.6 | 52.6 | 1650 | 0.2 | 7.0 |
| 7 | 1327 | 42.4 | 11.9 | 24.2 | 56.4 | 1399 | 42.2 | 9.0 | 28.7 | 52.6 | 1324 | 0.0 | 7.1 |
| 8 | 991 | 42.7 | 11.8 | 24.8 | 56.4 | 1047 | 42.1 | 9.0 | 28.8 | 52.5 | 989 | -0.1 | 7.0 |

## Table S6. Interactions between covariates and splines for SF-12 physical symptom scores

|  |  |  |  | **Females vs males** | | | **50-69 years vs 16-49 years** | | | **70+ years vs 16-49 years** | | | | **Low vs high deprivation** | | | | **Married/cohabiting vs not** | | |
| --- | --- | --- | --- | --- | --- | --- | --- | --- | --- | --- | --- | --- | --- | --- | --- | --- | --- | --- | --- | --- |
|  |  |  |  |  | | |  | | |  | | | |  | | | |  | | |
| PCS 8 years pre-arthritis | 54.17 | (52.44 | 55.89) | 0.09 | (-0.76 | 0.93) | -1.80 | (-2.90 | -0.70) | -3.66 | (-4.93 | -2.38) | 2.32 | | (1.41 | 3.24) | 0.72 | | (-0.26 | 1.69) |
| **Change in PCS** |  |  |  |  |  |  |  |  |  |  |  |  |  | |  |  |  | |  |  |
| 8 - 4 years pre-arthritis | -0.58 | (-0.96 | -0.21) | 0.10 | (-0.14 | 0.33) | -0.03 | (-0.32 | 0.26) | -0.04 | (-0.37 | 0.30) | 0.10 | | (-0.13 | 0.33) | 0.19 | | (-0.07 | 0.44) |
| 4 - 2 years pre-arthritis | -0.88 | (-1.41 | -0.34) | 0.06 | (-0.28 | 0.39) | 0.39 | (-0.04 | 0.82) | 0.17 | (-0.32 | 0.65) | -0.27 | | (-0.63 | 0.08) | 0.22 | | (-0.15 | 0.59) |
| 2 - 1 years pre-arthritis | -1.92 | (-2.85 | -0.99) | -0.36 | (-0.95 | 0.22) | 0.14 | (-0.61 | 0.89) | 0.10 | (-0.78 | 0.97) | 0.57 | | (-0.05 | 1.19) | 0.31 | | (-0.33 | 0.94) |
| 1 - 0 years pre-arthritis | -3.44 | (-4.38 | -2.50) | -0.47 | (-1.08 | 0.13) | 0.73 | (-0.01 | 1.48) | 0.68 | (-0.20 | 1.56) | -0.29 | | (-0.92 | 0.33) | -0.18 | | (-0.83 | 0.47) |
| 0 - 1 years post arthritis | 1.04 | (0.17 | 1.92) | 0.39 | (-0.18 | 0.96) | -0.16 | (-0.84 | 0.53) | -0.64 | (-1.48 | 0.21) | 0.34 | | (-0.25 | 0.93) | -0.21 | | (-0.82 | 0.40) |
| 1 - 4 years post arthritis | -0.10 | (-0.47 | 0.28) | 0.06 | (-0.18 | 0.29) | -0.31 | (-0.59 | -0.04) | -0.37 | (-0.73 | -0.01) | 0.13 | | (-0.12 | 0.37) | 0.19 | | (-0.07 | 0.46) |
| 4 -8 post arthritis | -0.07 | (-0.46 | 0.32) | -0.23 | (-0.50 | 0.03) | -0.20 | (-0.50 | 0.10) | -0.68 | (-1.10 | -0.25) | 0.09 | | (-0.18 | 0.36) | 0.04 | | (-0.26 | 0.33) |

N=5,118. Model additionally adjusted for ethnicity, education and ever having had a comorbidity at the time of arthritis diagnosis.

# GHQ-12 score models

The best fitting polynomial prior to arthritis diagnosis was 1 1 which uses the two terms time and time*ln(time). The best fitting polynomial after arthritis diagnosis -0.5 -0.5 which uses the two terms 1/sqrt(time) and 1/sqrt(time)*ln(time). The curves resulting from this analysis adjusted for age, sex, ethnicity, onset wave, education, ever having a comorbidity prior to diagnosis and deprivation category (IMD) are shown below.

**Figure S3. Best fitting polynomial before and after diagnosis for GHQ score**

## Table S7. Unadjusted spline models with 9 splines (N=5,220)

|  | Beta | 95% CI | |
| --- | --- | --- | --- |
| GHQ 8 years pre-arthritis | 11.75 | (11.50 | 12.00) |
| **Change in GHQ** |  |  |  |
| 8 - 6 years pre-arthritis | -0.06 | (-0.19 | 0.08) |
| 6 - 4 years pre-arthritis | 0.10 | (-0.01 | 0.22) |
| 4 - 2 years pre-arthritis | 0.07 | (-0.03 | 0.16) |
| 2 - 1 years pre-arthritis | 0.24 | (0.08 | 0.40) |
| 1 - 0 years post arthritis | 0.30 | (0.14 | 0.46) |
| 0 - 1 years post arthritis | -0.11 | (-0.27 | 0.05) |
| 1 - 4 years post arthritis | -0.00 | (-0.07 | 0.06) |
| 4 - 8 years post arthritis | 0.02 | (-0.05 | 0.09) |

Log likelihood = -137220.41

## Table S8. Unadjusted spline models with 5 splines (N=5,220)

|  | Beta | 95% CI | |
| --- | --- | --- | --- |
| GHQ 8 years pre-arthritis | 11.75 | (11.50 | 12.00) |
| **Change in GHQ** |  |  |  |
| 8 - 6 years pre-arthritis | -0.04 | (-0.17 | 0.09) |
| 6 - 2 years pre-arthritis | 0.08 | (0.03 | 0.13) |
| 2 - 0 years pre-arthritis | 0.26 | (0.18 | 0.35) |
| 0 - 1 years post arthritis | -0.11 | (-0.26 | 0.04) |
| 1 - 8 years post arthritis | 0.01 | (-0.03 | 0.04) |

Log likelihood = -137220.68

## Table S9. Adjusted spline models with 8 splines (N=5,178)

|  | Beta | 95% CI | |
| --- | --- | --- | --- |
| GHQ 8 years pre-arthritis | 12.25 | (11.40 | 13.10) |
| **Change in GHQ** |  |  |  |
| 8 - 6 years pre-arthritis | -0.05 | (-0.19 | 0.08) |
| 6 - 4 years pre-arthritis | 0.10 | (-0.02 | 0.21) |
| 4 - 2 years pre-arthritis | 0.07 | (-0.03 | 0.17) |
| 2 - 1 years pre-arthritis | 0.24 | (0.08 | 0.40) |
| 1 - 0 years post arthritis | 0.28 | (0.12 | 0.44) |
| 0 - 1 years post arthritis | -0.10 | (-0.26 | 0.06) |
| 1 - 4 years post arthritis | -0.00 | (-0.07 | 0.06) |
| 4 - 8 years post arthritis | 0.02 | (-0.05 | 0.09) |

Log likelihood = -135862.63

## Table S10. Adjusted spline models with 5 splines (N=5,178)

|  | Beta | 95% CI | |
| --- | --- | --- | --- |
| GHQ 8 years pre-arthritis | 12.25 | (11.40 | 13.10) |
| **Change in GHQ** |  |  |  |
| 8 - 6 years pre-arthritis | -0.04 | (-0.17 | 0.08) |
| 6 - 2 years pre-arthritis | 0.08 | (0.03 | 0.13) |
| 2 - 0 years pre-arthritis | 0.25 | (0.17 | 0.34) |
| 0 - 1 years post arthritis | -0.10 | (-0.25 | 0.05) |
| 1 - 8 years post arthritis | 0.01 | (-0.03 | 0.04) |

Log likelihood = -135862.8

## Figure S4. Residuals vs fitted values and normal probability plots for GHQ-12 scores at T=0, T=-8 and T=8

## T=0

**T=-8**

**T=8**

## Table S11. Predicted vs actual values for GHQ-12 scores

|  |  | **Observed values** | | | | **Fitted values** | | | | | **Difference**  **(Fitted -observed values)** | | |
| --- | --- | --- | --- | --- | --- | --- | --- | --- | --- | --- | --- | --- | --- |
| **Time since diagnosis (waves)** | **N** | **Mean** | **SD** | **90% limits of agreement** | | **N** | **Mean** | **SD** | **90% limits of agreement** | | **N** | **Mean** | **SD** |
| -8 | 1325 | 11.7 | 5.8 | 6 | 19 | 1500 | 11.6 | 4.1 | 7.3 | 17.2 | 1318 | -0.1 | 4.1 |
| -7 | 1685 | 11.4 | 5.6 | 6 | 19 | 1895 | 11.6 | 4.1 | 7.3 | 17.2 | 1676 | 0.1 | 3.9 |
| -6 | 1971 | 11.6 | 5.9 | 6 | 20 | 2215 | 11.6 | 4.2 | 7.3 | 17.4 | 1958 | 0.0 | 4.0 |
| -5 | 2383 | 11.6 | 5.9 | 6 | 20 | 2645 | 11.6 | 4.1 | 7.3 | 17.4 | 2371 | 0.0 | 4.1 |
| -4 | 2826 | 11.7 | 6.1 | 6 | 20 | 3088 | 11.8 | 4.1 | 7.4 | 17.5 | 2802 | 0.0 | 4.0 |
| -3 | 3267 | 11.7 | 5.9 | 6 | 20 | 3576 | 11.8 | 4.1 | 7.5 | 17.5 | 3241 | 0.0 | 4.1 |
| -2 | 3763 | 11.9 | 6.1 | 6 | 21 | 4136 | 11.9 | 4.1 | 7.5 | 17.6 | 3726 | 0.0 | 4.0 |
| -1 | 4429 | 12.1 | 6.1 | 6 | 21 | 4835 | 12.2 | 4.2 | 7.8 | 17.9 | 4385 | 0.0 | 3.9 |
| 0 | 4763 | 12.5 | 6.3 | 6 | 22 | 5105 | 12.5 | 4.2 | 8.1 | 18.3 | 4711 | 0.0 | 4.0 |
| 1 | 4031 | 12.3 | 6.2 | 6 | 21 | 4323 | 12.3 | 4.1 | 7.9 | 18.0 | 3992 | 0.0 | 4.1 |
| 2 | 3630 | 12.3 | 6.2 | 6 | 21 | 3831 | 12.3 | 4.1 | 7.9 | 18.0 | 3603 | -0.1 | 4.0 |
| 3 | 3184 | 12.0 | 6.0 | 6 | 21 | 3356 | 12.2 | 4.1 | 7.8 | 18.0 | 3163 | 0.1 | 3.9 |
| 4 | 2506 | 12.2 | 6.1 | 6 | 21 | 2635 | 12.2 | 4.1 | 7.8 | 17.8 | 2491 | 0.0 | 3.9 |
| 5 | 2085 | 12.2 | 6.1 | 6 | 21 | 2182 | 12.2 | 4.1 | 7.8 | 17.9 | 2072 | 0.0 | 4.1 |
| 6 | 1682 | 12.3 | 6.0 | 6 | 21 | 1753 | 12.3 | 4.1 | 7.8 | 18.1 | 1672 | 0.0 | 3.9 |
| 7 | 1344 | 12.0 | 5.9 | 6 | 21 | 1399 | 12.1 | 4.0 | 7.7 | 17.7 | 1341 | 0.0 | 4.1 |
| 8 | 1010 | 12.1 | 5.9 | 6 | 20 | 1046 | 12.1 | 4.1 | 7.7 | 17.7 | 1008 | -0.1 | 4.0 |

## Table S12. Interactions between covariates and splines for GHQ-12 scores

|  |  |  |  | **Females vs males** | | | **50-69 years vs 16-49 years** | | | **70+ years vs 16-49 years** | | | | **Low vs high deprivation** | | | | **Married/cohabiting vs not** | | |
| --- | --- | --- | --- | --- | --- | --- | --- | --- | --- | --- | --- | --- | --- | --- | --- | --- | --- | --- | --- | --- |
|  |  |  |  |  | | |  | | |  | | | |  | | | |  | | |
| GHQ 8 years pre-arthritis | 11.83 | (10.79 | 12.87) | 0.83 | (0.34 | 1.32) | -0.83 | (-1.58 | -0.08) | -2.86 | (-3.66 | -2.07) | -0.56 | | (-1.11 | -0.01) | -0.70 | | (-1.27 | -0.14) |
| **Change in GHQ** |  |  |  |  |  |  |  |  |  |  |  |  |  | |  |  |  | |  |  |
| 8 - 6 years pre-arthritis | -0.15 | (-0.62 | 0.33) | 0.01 | (-0.26 | 0.27) | 0.23 | (-0.17 | 0.63) | 0.18 | (-0.22 | 0.59) | -0.06 | | (-0.35 | 0.23) | -0.05 | | (-0.35 | 0.25) |
| 6 - 2 years pre-arthritis | 0.20 | (0.02 | 0.39) | 0.04 | (-0.07 | 0.14) | -0.14 | (-0.29 | 0.02) | -0.10 | (-0.26 | 0.06) | 0.03 | | (-0.09 | 0.14) | -0.09 | | (-0.21 | 0.03) |
| 2 - 0 years pre-arthritis | 0.53 | (0.25 | 0.81) | -0.14 | (-0.30 | 0.03) | -0.38 | (-0.62 | -0.13) | -0.24 | (-0.49 | 0.02) | 0.01 | | (-0.17 | 0.20) | 0.07 | | (-0.13 | 0.26) |
| 0 - 1 years post arthritis | -0.50 | (-1.02 | 0.02) | 0.23 | (-0.07 | 0.53) | -0.04 | (-0.46 | 0.37) | 0.09 | (-0.34 | 0.52) | -0.19 | | (-0.52 | 0.13) | 0.55 | | (0.20 | 0.90) |
| 1 - 8 years post arthritis | -0.05 | (-0.18 | 0.07) | 0.04 | (-0.03 | 0.12) | -0.02 | (-0.12 | 0.07) | 0.08 | (-0.03 | 0.18) | 0.07 | | (-0.01 | 0.15) | -0.02 | | (-0.11 | 0.07) |

N=5,098. Model additionally adjusted for ethnicity, education and ever having had a comorbidity at the time of arthritis diagnosis.

# Health Satisfaction models

The best fitting polynomial prior to arthritis diagnosis was -0.5 0 which uses the two terms 1/sqrt(time) and ln(time). The best fitting polynomial after arthritis diagnosis was -0.5 -0.5 which uses the two terms 1/sqrt(time) and 1/sqrt(time) *ln(time). The curves resulting from this analysis adjusted for age, sex, ethnicity, onset wave, education, ever having a comorbidity prior to diagnosis and deprivation category (IMD) are shown below.

## Figure S5. Best fitting polynomial before and after diagnosis for Health Satisfaction

## Table S13. Unadjusted spline models with 9 splines (N=5,217)

|  | Beta | 95% CI | |
| --- | --- | --- | --- |
| 8 years pre-arthritis | 4.48 | (4.41 | 4.55) |
| **Change in Health Satisfaction** |  |  |  |
| 8 - 4 years pre-arthritis | 0.00 | (-0.02 | 0.02) |
| 4 - 2 years pre-arthritis | -0.03 | (-0.06 | -0.00) |
| 2 - 1 years pre-arthritis | -0.11 | (-0.16 | -0.05) |
| 1 - 0 years pre-arthritis | -0.24 | (-0.29 | -0.18) |
| 0 - 1 years post arthritis | 0.09 | (0.04 | 0.15) |
| 1 - 2 years post arthritis | 0.00 | (-0.06 | 0.06) |
| 2 - 3 years post arthritis | 0.09 | (0.03 | 0.15) |
| 3 - 4 years post arthritis | -0.03 | (-0.09 | 0.03) |
| 4 -8 years post arthritis | 0.01 | (-0.01 | 0.03) |

Log likelihood = -83373.475

## Table S14. Unadjusted spline models with 5 splines (N=5,239)

|  | Beta | 95% CI | |
| --- | --- | --- | --- |
| 8 years pre-arthritis | 4.50 | (4.44 | 4.56) |
| **Change in Health Satisfaction** |  |  |  |
| 8 - 2 years pre-arthritis | -0.01 | (-0.02 | 0.00) |
| 2 - 1 years pre-arthritis | -0.13 | (-0.18 | -0.08) |
| 1- 0 years pre-arthritis | -0.23 | (-0.28 | -0.18) |
| 0 - 3 years post arthritis | 0.05 | (0.03 | 0.07) |
| 3 - 8 years post arthritis | 0.00 | (-0.01 | 0.02) |

Log likelihood = -83376.528

## Table S15. Adjusted spline models with 9 splines (N= 5,156)

|  | Beta | 95% CI | |
| --- | --- | --- | --- |
| 8 years pre-arthritis | 4.26 | (4.05 | 4.47) |
| **Change in Health Satisfaction** |  |  |  |
| 8 - 4 years pre-arthritis | 0.00 | (-0.02 | 0.02) |
| 4 - 2 years pre-arthritis | -0.03 | (-0.06 | -0.00) |
| 2 - 1 years pre-arthritis | -0.11 | (-0.17 | -0.06) |
| 1 - 0 years pre-arthritis | -0.23 | (-0.28 | -0.18) |
| 0 - 1 years post arthritis | 0.09 | (0.03 | 0.14) |
| 1 - 2 years post arthritis | -0.00 | (-0.06 | 0.06) |
| 2 - 3 years post arthritis | 0.09 | (0.03 | 0.15) |
| 3 - 4 years post arthritis | -0.03 | (-0.08 | 0.03) |
| 4 -8 years post arthritis | 0.01 | (-0.01 | 0.03) |

Log likelihood = -82553.102

## Table S16. Adjusted spline models with 5 splines (N=5,156)

|  | Beta | 95% CI | |
| --- | --- | --- | --- |
| 8 years pre-arthritis | 4.28 | (4.07 | 4.49) |
| **Change in Health Satisfaction** |  |  |  |
| 8 - 2 years pre-arthritis | -0.01 | (-0.02 | 0.00) |
| 2 - 1 years pre-arthritis | -0.13 | (-0.18 | -0.08) |
| 1- 0 years pre-arthritis | -0.22 | (-0.27 | -0.17) |
| 0 - 3 years post arthritis | 0.05 | (0.03 | 0.07) |
| 3 - 8 years post arthritis | 0.00 | (-0.01 | 0.02) |

Log likelihood = -82556.275

## Figure S6. Residuals vs fitted values and normal probability plots for Health Satisfaction scores at T=0, T=-8 and T=8

**T=0**

**T=-8**

**T=8**

## Table S17. Predicted vs actual values for Health Satisfaction

|  |  | **Observed values** | | | | **Fitted values** | | | | | **Difference**  **(Fitted -observed values)** | | |
| --- | --- | --- | --- | --- | --- | --- | --- | --- | --- | --- | --- | --- | --- |
| **Time since diagnosis (waves)** | **N** | **Mean** | **SD** | **90% limits of agreement** | | **N** | **Mean** | **SD** | **90% limits of agreement** | | **N** | **Mean** | **SD** |
| -8 | 1326 | 4.6 | 1.7 | 2 | 6 | 1500 | 4.6 | 1.0 | 3.2 | 5.8 | 1319 | 0.0 | 1.3 |
| -7 | 1677 | 4.5 | 1.7 | 2 | 6 | 1895 | 4.5 | 1.0 | 3.2 | 5.8 | 1670 | 0.0 | 1.4 |
| -6 | 1963 | 4.5 | 1.7 | 2 | 6 | 2215 | 4.5 | 1.0 | 3.2 | 5.8 | 1953 | 0.0 | 1.4 |
| -5 | 2390 | 4.5 | 1.7 | 2 | 6 | 2645 | 4.5 | 1.0 | 3.1 | 5.7 | 2378 | 0.0 | 1.4 |
| -4 | 2795 | 4.5 | 1.7 | 2 | 6 | 3089 | 4.5 | 1.0 | 3.1 | 5.7 | 2784 | 0.0 | 1.3 |
| -3 | 3257 | 4.5 | 1.7 | 2 | 6 | 3578 | 4.5 | 1.0 | 3.1 | 5.7 | 3234 | 0.0 | 1.3 |
| -2 | 3770 | 4.4 | 1.7 | 2 | 6 | 4137 | 4.5 | 1.0 | 3.1 | 5.7 | 3738 | 0.0 | 1.3 |
| -1 | 4417 | 4.3 | 1.7 | 2 | 6 | 4834 | 4.3 | 1.0 | 3.0 | 5.6 | 4386 | 0.0 | 1.3 |
| 0 | 4790 | 4.1 | 1.7 | 2 | 6 | 5104 | 4.1 | 1.0 | 2.7 | 5.3 | 4736 | 0.0 | 1.2 |
| 1 | 4049 | 4.2 | 1.7 | 2 | 6 | 4322 | 4.2 | 1.0 | 2.8 | 5.4 | 4009 | 0.0 | 1.3 |
| 2 | 3651 | 4.2 | 1.7 | 2 | 6 | 3831 | 4.2 | 1.0 | 2.8 | 5.5 | 3624 | 0.0 | 1.2 |
| 3 | 3200 | 4.3 | 1.7 | 2 | 6 | 3356 | 4.3 | 1.0 | 2.9 | 5.5 | 3178 | 0.0 | 1.2 |
| 4 | 2521 | 4.3 | 1.7 | 2 | 6 | 2635 | 4.3 | 1.0 | 2.9 | 5.5 | 2505 | 0.0 | 1.2 |
| 5 | 2096 | 4.3 | 1.7 | 2 | 6 | 2182 | 4.3 | 1.0 | 2.9 | 5.6 | 2083 | 0.0 | 1.2 |
| 6 | 1688 | 4.3 | 1.7 | 2 | 6 | 1753 | 4.3 | 1.0 | 2.9 | 5.6 | 1677 | 0.0 | 1.2 |
| 7 | 1353 | 4.4 | 1.7 | 2 | 6 | 1399 | 4.3 | 1.0 | 2.9 | 5.6 | 1350 | 0.0 | 1.3 |
| 8 | 1014 | 4.3 | 1.7 | 2 | 6 | 1046 | 4.3 | 1.0 | 3.0 | 5.6 | 1012 | 0.0 | 1.2 |

## Table S18. Interactions between covariates and splines for Health Satisfaction

|  |  |  |  | **Females vs males** | | | **50-69 years vs 16-49 years** | | | **70+ years vs 16-49 years** | | | | **Low vs high deprivation** | | | | **Married/cohabiting vs not** | | |
| --- | --- | --- | --- | --- | --- | --- | --- | --- | --- | --- | --- | --- | --- | --- | --- | --- | --- | --- | --- | --- |
|  |  |  |  |  | | |  | | |  | | | |  | | | |  | | |
| 8 years pre-arthritis | 4.62 | (4.38 | 4.86) | 0.06 | (-0.07 | 0.19) | -0.07 | (-0.23 | 0.09) | 0.41 | (0.23 | 0.59) | 0.19 | | (0.06 | 0.33) | 0.17 | | (0.04 | 0.31) |
| **Change in Health Satisfaction** |  |  |  |  |  |  |  |  |  |  |  |  |  | |  |  |  | |  |  |
| 8 - 2 years pre-arthritis | -0.04 | (-0.08 | 0.00) | -0.00 | (-0.03 | 0.02) | 0.04 | (0.01 | 0.07) | 0.02 | (-0.02 | 0.05) | 0.00 | | (-0.02 | 0.03) | 0.01 | | (-0.02 | 0.03) |
| 2 - 1 years pre-arthritis | -0.26 | (-0.43 | -0.10) | -0.09 | (-0.20 | 0.02) | 0.11 | (-0.02 | 0.25) | 0.21 | (0.06 | 0.36) | 0.03 | | (-0.08 | 0.14) | 0.09 | | (-0.02 | 0.20) |
| 1- 0 years pre-arthritis | -0.30 | (-0.46 | -0.14) | 0.08 | (-0.02 | 0.18) | 0.06 | (-0.07 | 0.18) | 0.01 | (-0.13 | 0.16) | 0.04 | | (-0.06 | 0.15) | -0.05 | | (-0.16 | 0.06) |
| 0 - 3 years post arthritis | 0.07 | (0.02 | 0.13) | -0.02 | (-0.06 | 0.02) | 0.02 | (-0.02 | 0.06) | -0.01 | (-0.06 | 0.04) | -0.03 | | (-0.06 | 0.01) | -0.00 | | (-0.04 | 0.04) |
| 3 - 8 years post arthritis | 0.02 | (-0.02 | 0.07) | 0.00 | (-0.03 | 0.03) | -0.03 | (-0.06 | 0.01) | -0.06 | (-0.11 | -0.02) | 0.01 | | (-0.02 | 0.04) | 0.00 | | (-0.03 | 0.04) |

N=5,097. Model additionally adjusted for ethnicity, education and ever having had a comorbidity at the time of arthritis diagnosis.

## Figure S7. Trajectories restricted to individuals with data at all waves (1-13) of UKHLS

A

B

C

A.SF-12 Physical symptom score, B. GHQ score, C. Health Satisfaction

## Table S19. Spline models for physical component score, GHQ-12 and health satisfaction restricted to individuals with data in all waves

| **SF-12 Physical Symptom Score** | | | |
| --- | --- | --- | --- |
| **N=3,098** | Beta | 95% CI | |
| PCS 8 years pre-arthritis | 54.70 | (51.93 | 57.47) |
| **Change in PCS** |  |  |  |
| 8 - 4 years pre-arthritis | -0.27 | (-0.53 | -0.01) |
| 4 - 2 years pre-arthritis | -0.63 | (-1.04 | -0.21) |
| 2 - 1 years pre-arthritis | -1.41 | (-2.04 | -0.78) |
| 1 - 0 years pre-arthritis | -3.60 | (-4.34 | -2.86) |
| 0 - 1 years post arthritis | 0.82 | (0.17 | 1.47) |
| 1 - 4 years post arthritis | -0.26 | (-0.53 | 0.02) |
| 4 -8 post arthritis | -0.52 | (-0.83 | -0.21) |

| **GHQ-12** | | | |
| --- | --- | --- | --- |
| **N=3,097** | Beta | 95% CI | |
| GHQ 8 years pre-arthritis | 10.75 | (9.05 | 12.44) |
| **Change in GHQ** |  |  |  |
| 8 - 6 years pre-arthritis | 0.05 | (-0.22 | 0.32) |
| 6 - 2 years pre-arthritis | -0.05 | (-0.18 | 0.08) |
| 2 - 0 years pre-arthritis | 0.33 | (0.11 | 0.56) |
| 0 - 1 years post arthritis | -0.03 | (-0.41 | 0.47) |
| 1 - 8 years post arthritis | 0.01 | (-0.08 | 0.11) |

| **Health Satisfaction** | | | |
| --- | --- | --- | --- |
| **N=3,097** | Beta | 95% CI | |
| 8 years pre-arthritis | 4.41 | (4.00 | 4.81) |
| **Change in Health Satisfaction** |  |  |  |
| 8 - 2 years pre-arthritis | -0.02 | (-0.05 | 0.01) |
| 2 - 1 years pre-arthritis | -0.08 | (-0.21 | -0.05) |
| 1- 0 years pre-arthritis | -0.24 | (-0.36 | -0.11) |
| 0 - 3 years post arthritis | 0.03 | (-0.02 | 0.07) |
| 3 - 8 years post arthritis | -0.03 | (-0.07 | 0.01) |

## Table S20. Differences in SF-12 physical component score, GHQ-12 and health satisfaction between arthritis cases and controls 8 years prior to diagnosis

|  | **N** | **Beta**  **(Arthritis case – control)** | **95% CI** | **P-value** |
| --- | --- | --- | --- | --- |
| SF-12 Physical component score | 2,202 | -1.87 | (-2.68, -1.06) | <0.001 |
| GHQ-12 | 2,247 | 0.67 | (0.21, 1.12) | 0.004 |
| Health Satisfaction | 2,257 | -0.26 | (-0.40, -0.13) | <0.001 |

Adjusted for age, sex, education, Indices of multiple deprivation, ethnicity, any comorbidity ever
